# Uncovering the Basis of ATP Hydrolysis Activity in Purified Human p53 Protein: A Reinvestigation

**DOI:** 10.1371/journal.pone.0093652

**Published:** 2014-04-01

**Authors:** Shalini Verma, Basuthkar J. Rao

**Affiliations:** Department of Biological Sciences, Tata Institute of Fundamental Research, Mumbai, Maharashtra, India; University of South Florida College of Medicine, United States of America

## Abstract

p53 is one of the most well studied tumor suppressor proteins and regarded as the guardian of the genome. The protein mediates cell-cycle arrest, apoptosis in response to myriads of cellular stresses including DNA damage via its trascriptional as well as non-transcriptional roles. ATP binding/hydrolysis by p53 had been implicated in its DNA binding functions. However, till date, no ATP binding/hydrolysis domains have been mapped in p53. In the current study, we have reinvestigated the ATP hydrolysis activity associated with recombinant human p53 protein expressed and purified from *E.coli*. We confirmed the source of ATPase activity using various deletion constructs of p53 and an In-gel ATPase assay followed by LC-ESI-MS/MS analysis of the activity band. The activity was associated with Hsp70 homologue in *E.coli*, DnaK, a known interactor of p53. We clarify that wildtype human p53, expressed in *E. coli* BL21 (DE3) strain, carries no ATPase activity.

## Introduction

p53, also known as the guardian of genome [1], is the most well studied tumor suppressor protein. The p53 gene has found to be frequently mutated in most human cancers [2]. p53 null mice can develop normally but nearly all develop cancer before 6 months of age [3]. p53 levels are maintained low in the cell under normal physiological condition by E3 ligase Mdm2 which targets p53 for ubiquitination and proteasome mediated degradation [4]. Under various stresses including DNA damage, p53 levels increase in the cell, as the p53-Mdm2 complex dissociates [5]. p53 accumulates and gets stabilized by rapid post translational modifications including phosphorylation, methylation, acetylation, sumoylation and glycosylation [6]. Upon localization to the nucleus, p53 functions as a transcription factor where it can activate or repress the transcription of many downstream target genes involved in cellular responses to stress, such as cell cycle arrest, DNA repair, senescence and apoptosis [7]. p53 suppresses tumorigenesis by preventing propagation and transmission of damaged DNA with potentially harmful mutations.

p53 is well known to bind to the specific sequence, p53 response element (p53RE) present in the promoter regions of p53 target genes [8]. Consensus sequence of p53RE comprises of a 10 bp palindromic sequence made up of two ‘half sites’—PuPuPu C (A/T)(T/A) G PyPyPy (*n*) PuPuPu C (A/T)(T/A) G PyPyPy separated by a spacer of 0–13 bases (*n*) [8], [9]. 393 amino acids protein, p53 binds to the p53RE through its DNA binding domain (102–292 amino acids) which is a hot-spot for mutations in p53 for majority of the human cancers [10]. Previous studies implicated ATP binding to the C terminus of p53 [11] modulating the release of p53 from p53-DNA complex [12], [13]. Indirect evidence suggested that ATP:ADP ratio influences the conformation of p53 protein [14]. More than a decade ago, Okorokov *et al*. showed that p53 not only binds ATP, but can also hydrolyze it [15] and suggested that human p53 converts ATP to ADP, creating ADP bound form of p53 for stable DNA binding. This proposal is in line with the modulation of DNA binding affinity upon ATP binding/hydrolysis as observed with other proteins such as hRAD51 and *E.coli* RecA [16], [17], [18]. However, it is also relevant to point out here that there has been no direct evidence of an unequivocal site(s) of ATP binding in p53 till date, neither the ATP hydrolysis domain has been mapped, nor the ATP hydrolysis mutants of p53 have been generated. No physiological functions have been assigned to the ATP binding and hydrolysis activity of p53. Therefore, it is fair to say that the putative role of ATP binding and its hydrolysis by p53 remains largely unsubstantiated in the context of known biology of p53 protein. In this study we further investigated the ATPase activity associated with purified p53 protein. We believe that the current study provides an insight into the impasse related to the ascribed activity of ATP binding/hydrolysis in p53 protein.

## Materials and Methods

### Protein purification

#### Full length human p53

The protein was expressed in *E.coli* BL21(DE3) transformed with pET28a-GST vector containing human p53 gene (kind gift from Jörg Kobarg, CBME, Brazil). The transformed cells, grown at 37°C till A_600_ of 0.5 in LB medium containing 50 μg/ml kanamycin, were induced with 0.5 mM IPTG at 25°C and harvested after 12 hours. The cells were resuspended in 25 mM HEPES-KOH (pH 7.6), 0.1 M KCl, 2 mM EDTA, 2 mM DTT, 20% glycerol, 1 mM Benzamidine, 0.25 mM PMSF and protease inhibitors cocktail (Roche), incubated with lysozyme (1 mg/ml) on ice for 30 minutes and sonicated after adding 0.1% NP-40. The cell lysate was centrifuged at 18,000 rpm for 45 minutes at 4°C. The supernatant was diluted five times in volume with 50 mM NaH_2_PO_4_ (pH 8.0), 1 mM DTT, 1 mM Benzamidine, 0.1 mM PMSF and protease inhibitors cocktail (Roche), followed by incubation with pre-equilibriated Glutathione S sepharose beads (GE Healthcare) for 2 hours at 4°C. The beads were then packed into an Econo-column (Bio-Rad Laboratories). The resin was washed with 50 mM NaH_2_PO_4_ (pH 8.0), 0.3 M KCl, 1 mM DTT, 1 mM Benzamidine and 0.1 mM PMSF. The protein was eluted with 20 mM reduced glutathione in 50 mM NaH_2_PO_4_ (pH 8.0), 0.3 M KCl, 1 mM DTT, 1 mM Benzamidine and 0.1 mM PMSF and dialyzed against 40 mM NaH_2_PO_4_ (pH 8.0), 50 mM KCl, 2 mM DTT and 5% glycerol. The dialysed protein was stored at −80°C. The dialysed protein was further purified by FPLC-gel filtration (size exclusion) chromatography using GE healthcare AKTA system and HiLoad 16/60 Superdex 200 pg. The flow rate was maintained at 1 ml per minute. The protein fractions were eluted in buffer containing 40 mM NaH_2_PO_4_ (pH 8.0), 50 mM KCl, 2 mM DTT and 5% glycerol. 120 fractions (1 ml/fraction) were collected in 2 hours. The equal volume of peak fractions were analysed for ATP hydrolysis activity.

Similarly, the GST tagged full length p53 was expressed and purified from ΔDnaK BL21(DE3) *E.coli* cells (kind gift from Dr. Pierre Genevaux, CNRS, France), except that the cells were cultured at 30°C instead of 37°C till 0.6 O.D., as the cells are temperature sensitive.

#### Human p53 deletion mutants

Clones 3C, 24, 25 and 35, inserted into the expression vector pET11GST (kind gift from Prof. Bruce Stillman, Cold Spring Harbor Laboratory) encode GST fused to amino acids 155–393, 94–269, 94–293 and 155–299 of p53, respectively. All the deletion constructs were purified as per the protocol followed for full length wildtype p53-GST (described above), except the cultures were grown in LB media containing 100 μg/ml ampicillin instead of kanamycin.

Proteins were analyzed by SDS-PAGE [19] and concentrations were determined using the Bradford method, with bovine serum albumin as the standard protein.

### Immunoblotting

The p53 protein was subjected to electrophoresis on a 12% SDS polyacrylamide gel and was transferred to Immun-Blot PVDF membrane (Bio-Rad Laboratories). The primary antibody used to probe the blot in this study was mouse monoclonal anti p53 pAb122 (sc-56182; Santa Cruz biotechnology, inc.). The antibody was diluted 1∶3000 in 1% casein in 1× TBST solution and incubated overnight at 4°C. Horseradish peroxidise linked secondary antibody was anti mouse IgG (Roche diagnostics). The secondary antibody was diluted 1∶2000 in 1% casein in 1× TBST solution and incubated for 1 hour at room temperature. The blot was incubated with SuperSignal West Dura (Thermo Scientific) chemiluminescent substrate. The blot was exposed to Super Rx blue sensitive X-ray film (Fujifilm) and developed using Optimax 2010 X-ray film processor.

### ATPase Assay

NADH coupled microtiter plate assay was performed as described in published protocol [20]. ATPase assay time-course was performed with varying proteins concentrations (as indicated in figure legends) up to 180 minutes at 30°C in ATPase buffer (described in [20]) with 1 mM NADH. NADH absorbance decline was measured at 340 nm in 96 well plate using Tecan Infinite M-200 spectrophotometer. ATPase buffer in the absence of proteins was taken as the negative control. ATPase buffer containing 5 mM ADP (Roche) was taken as positive control to verify the activity of coupled reaction.

Radioactive ATPase assay was performed using thin layer chromatography (TLC) with 2 μM each of GST tagged full length wildtype p53 (p53-FL), p53-3C, p53-24, p53-25 or p53-35 incubated with 0.25 μM of 50 nCi/μl [α-^32^P] ATP (BRIT, Hyderabad, India) in 25 mM Tris–HCl (pH 7.6), 1.8 mM DTT and 13 mM magnesium acetate at 30°C for 30 minutes. The reactions were stopped with 0.2% SDS, heated at 100°C for 3 minutes and spotted on polyethyleneimine sheet (Merck) that was developed in 0.75 M KH_2_PO_4_ (pH 3.6). The dried PEI sheet was scanned using Molecular Dynamics Storm 820 PhosphorImager. The percentage of [α-^32^P] ATP hydrolyzed to [α-^32^P] ADP was quantified by Image J software.

### In-gel ATPase assay

Purified human p53 protein was treated with AcTEV protease (Invitrogen) using manufacturer's protocol at 30°C for 2 hours, wherever mentioned. TEV treated and untreated p53 protein samples resuspended in 1× Laemmli buffer with reduced SDS (1.25% instead of 2.5%) without heating and was electrophoresed on SDS-PAGE (12%) gel containing reduced SDS (0.05% instead of 0.1%) in both stacking and resolving portions using SDS-PAGE buffer with reduced SDS (0.05% instead of 0.1%). After electrophoresis, the gel was either stained with coomassie brilliant blue stain or analysed for ATPase activity using a modified protocol from [21]. The protein was allowed to recover the conformation by incubating the gel in Buffer A [35 mM TrisCl (pH 8.0), 270 mM Glycine, 14 mM MgSO_4_, 5 mM ATP] containing 50% isopropanol at 30°C for an hour with mild shaking to remove SDS. The gel is then incubated at 30°C for about 24 hrs in Buffer A containing 20% methanol and 0.075% lead nitrate till the white ATPase activity band appeared as a result of lead phosphate precipitate formation within the gel. The buffer was changed after 12 hrs in between the incubation. The ATPase activity stained band was excised and prepared for LC-ESI-MS/MS following trypsin digestion.

### LC-ESI-MS/MS analysis

For the preparation of samples for mass spectrometric analysis, the gel was serially sliced, minced and digested with trypsin and peptides were extracted [22]. Peptides extracted from trypsin treated samples were analyzed by LC-ESI-MS/MS using an Agilent 6520-QTOF. Peptides were taken in 3 μl of 0.1% formic acid (FA) (Solvent A). Typically, 2 μl of this sample was applied to an Agilent HPLC chip (G4240-62002). Nano-chip comprised of a 40 nl enrichment column and a 75 μm×150 mm separation column that was packed with Zorbax 300 SB-C18 (5 μm) material. After sample injection, the column was washed for 2 min with 0–3% Solvent B (90% acetonitrile in 0.1% formaldehyde), and peptides were eluted for 2–6 min with 1–30%, 6–15 min with 30–70%, 15–20 min with 70–95% Solvent B. Active exclusion was set-on for 0.5 min after each MS/MS spectrum.The m/z range used was 100–1700 for MS and 50–1700 for MS/MS. MS and MS/MS scan rate was 1.36 per second. For each MS, five most abundant precursor ions were sequenced.The mgf files were generated in MassHunter workstation software. The mgf files so obtained were submitted for protein identification searches against ‘*H.sapien* and *E.coli* databases from NCBInr’ using an in-house Mascot server.

## Results and Discussion

We have studied the effect of p53 and BRC repeats domain of BRCA2, BRCA2_BRC1-8_ on hRAD51 mediated ATP hydrolysis [23]. As a control, we used ATP hydrolysis reaction containing only p53 protein, where we also detected ATPase activity with purified p53. In this paper, we further investigated the basis of ATPase activity associated with recombinant human p53 protein, using a systematic approach.

**Figure 1:**
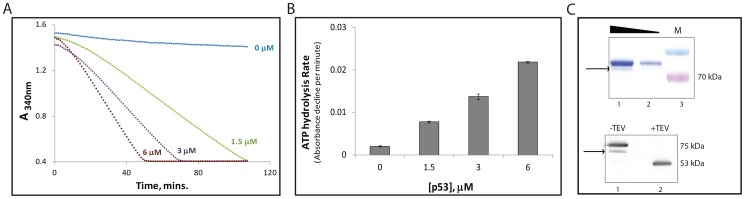
Concentration dependent increase in ATP hydrolysis by human p53 protein. (A) Using the real time assay, we studied ATPase activity of GST tagged recombinant human p53. Graph shows decline in absorbance at 340 nm as a function of time. The absorbance decline accounts for NADH decomposition which is a measurable read-out for ATPase reaction. The ATPase rate measured as a slope of the data points, increases with increase in the protein concentration. Varying protein concentrations are mentioned with same color as the data points. Negative control (0 μM p53) contained 5 μM GST-tag without p53 showing spontaneous NADH decomposition. (B) ATPase rates of p53 calculated as the slopes of data points in (A) plotted as a function of increasing protein concentration. The experiment was done in triplicates and repeated thrice. The graphs shown in (A) are a representative set. Error bars in (B) indicate the standard deviation across triplicates of three independent experiments. (C) GST tagged affinity purified p53 protein analyzed on coomassie stained SDS-PAGE gel at concentrations, 5 μg (lane 1) and 1 μg (lane 2) in upper panel. Lower panel shows the purified protein p53-GST immunoblotted against C-terminus of p53 with (Lane 2) and without (Lane 1) TEV protease treatment that cleaves away the N-terminal GST tag from p53. The arrows in both the panels indicate the minor band which comprises of degraded p53.

We expressed and purified the N-terminal GST tagged human p53 from *E.coli* BL21(DE3). Using NADH-coupled ATPase assay, we detected ATP hydrolysis whose rates increased as a function of p53 concentration. This real-time assay showed distinct slopes of absorbance decline as a function of reaction time that revealed the rates of ATPase reactions (Figure 1A–B). The ATPase activity recovered from p53 preparation was not due to associated GST tag, as purified GST-tag itself did not carry any ATPase activity (data not shown). Moreover, even Histidine-tagged p53 protein carried the same level of ATPase as the GST-tagged version. In order to assess the homogeneity of purified p53, we analysed the protein by SDS polyacrylamide gel electrophoresis. We observed the presence of minor bands below the full length p53-GST in the coomassie stained gel (lane 1, upper panel, Figure 1C). Western blotting using anti p53 C-terminus antibody, displayed a single faint band below the prominent full length p53-GST band (lower panel, Figure 1C) overlapping with the minor band indicated by an arrow in the coomassie stained gel.

To test if the minor band(s) observed in coomassie stained gel are clipped products of p53 or not, we cleaved GST tag in p53-GST using TEV protease before performing immunostaining with anti p53 C-terminus antibody. A single p53 band at ∼53 kDa position was evident on the western blot (Lane 2, lower panel, Figure 1C). We therefore infer that the faint western positive band in lane 1 was a partially clipped N-terminal GST tag in p53-GST protein. p53-GST and its N-terminal clipped form were converted to GST-free full length p53 protein following TEV protease treatment, revealing as a single western positive band (lane 2, lower panel, Figure 1C).

**Figure 2:**
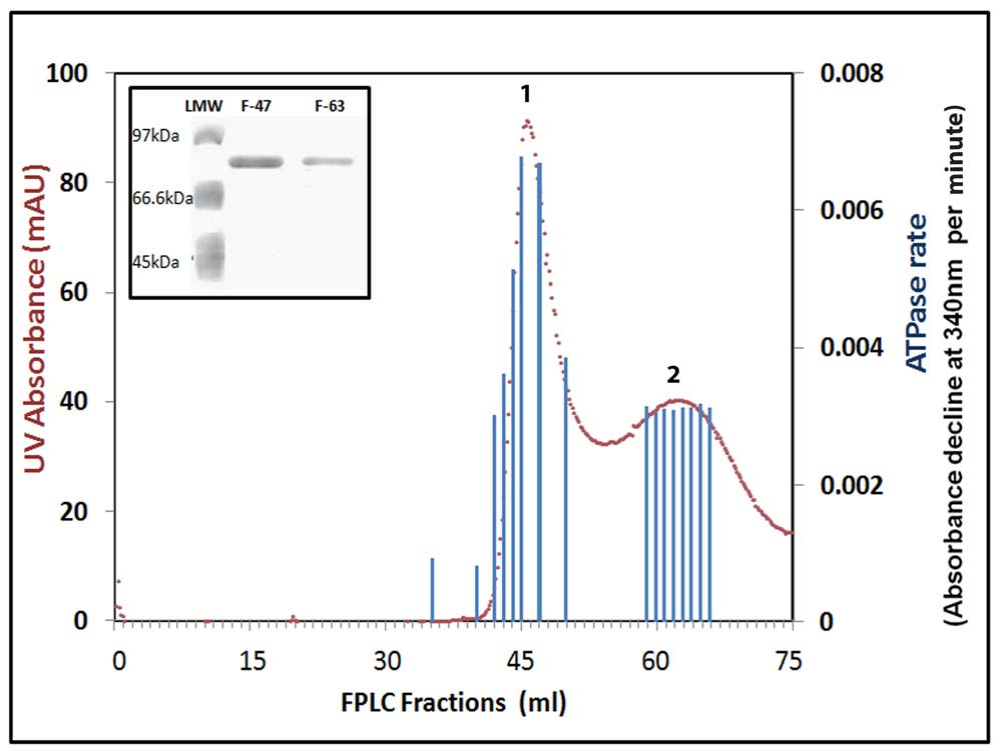
ATP hydrolysis activity corresponds to p53 peaks in FPLC gel filtration profile. The FPLC profile of p53 shows two peaks for UV absorbance at 280(in Red) corresponding to higher oligomeric form (peak 1) and monomeric form (peak 2). The peak fractions 47 and 63 were loaded on SDS-PAGE gel and both show the same size band corresponding to p53-GST (***Inset***). Equal volume of various fractions collected from FPLC elution was taken to perform NADH dependent ATP hydrolysis assay. The rates of ATP hydrolysis (Absorbance decline at 340 nm per min) are plotted on the secondary Y axis as bar graph (in Blue) to compare the protein concentration and ATPase activity for each fraction.

We performed FPLC gel filtration chromatography on the affinity purified GST tagged p53. Two distinct peaks were observed, where calibration by standard markers showed that the second peak corresponds to the monomer state of p53 and the first one to a higher oligomerization state (Figure 2). The peak fractions 47 and 63, were analyzed on the SDS-PAGE gel. Both the fractions showed single band at the same position corresponding to p53 (Figure 2, inset). We analysed ATP hydrolysis in various fractions by taking equal volume aliquots. We found that the computed ATPase rates were proportional to protein absorbance (Figure 2), thereby revealing that ATPase activity might be associated with p53 protein.

**Figure 3:**
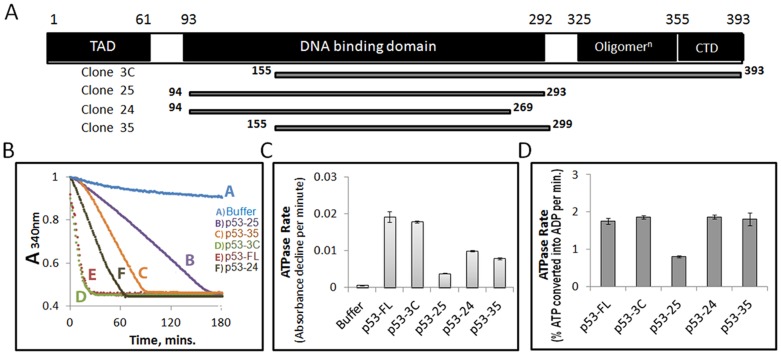
Deletion mutants of p53 and their ATPase activities. (A) Schematic drawing indicates the functional domains in full-length p53 (p53-FL) and the portions that are present in the deletion mutants. The numbers denote amino acid residues. (B) Using the real time assay, we compared ATPase activity of recombinant full length wild type and mutant forms of human p53. All the p53 proteins were taken at equal concentration of 5 μM. (C) Graph shows the ATPase rates of the full length and mutant forms of p53. ATPase rates were calculated as slopes of the plots in Figure (B). (D) Rates of ATP hydrolysis by p53 full-length wild type and deletion mutants from radioactive TLC assay, performed to corroborate the real time assay results. ATPase rates were calculated as the percentage of ATP converted into ADP per minute. The graphs shown in (B) are a representative set from three independent experiments. Error bars in (C) – (D) indicate the standard deviation across triplicates of three independent experiments.

In order to further validate the ATPase activity of p53 protein, we analyzed the ATPase rates of various p53 deletion mutants. Figure 3A provides a schematic representation of these mutants. Mutant clone 3C showed ATPase rate equivalent to the full length p53 (Figure 3B–C). It appears that the first 154 amino acid residues can be dispensed without losing any ATPase function. However, C-terminal deletions affected the ATPase rates: Clone 25 was least active in ATP hydrolysis. Surprisingly, a sub-deletion of clone 25 (clone 24) showed improved ATPase. Clone 24 showed substantial improvement in ATPase rate compared to clone 25, whereas clone 35 activity was intermediate between that of clones 24 and 25 (Figure 3B–C). To further corroborate these results, we also used conventional radioactive TLC assay with [α^32^P] ATP (Figure 3D). Mutant clone 25 showed the least ATPase rate as in coupled assay. However, the difference between the rates of full length wildtype protein/clone 3C, clones 24 and 35 diminished due to lower sensitivity of radioactive assay compared to real time NADH coupled assay.

**Figure 4:**
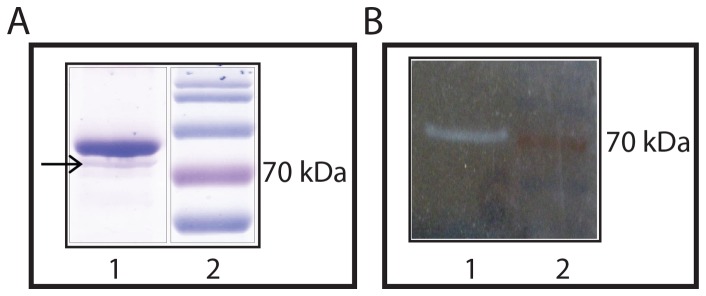
Mapping the ATPase activity in the purified human p53 protein using In-gel assay following SDS-PAGE. (A) 15 μg of purified p53-GST protein was resolved on SDS-PAGE (12%) gel and stained with coomassie brilliant blue. The GST tagged full length p53 is observed at ∼75 kDa position with a lower molecular weight minor band just below. (B) The In-gel ATPase assay for 15 μg of purified p53-GST protein showing a white band due to the formation of lead phosphate upon ATP hydrolysis (Lane 1). The activity band aligns with the minor band indicated by an arrow in (A).

**Figure 5:**
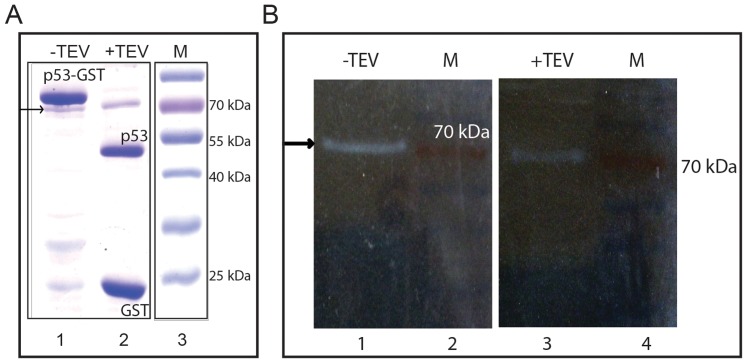
In-gel ATPase assay after removal of GST tag from p53 protein by TEV protease treatment. (A) Coomassie stained SDS-PAGE showing p53-GST protein with (Lane 2) and without (Lane 1) TEV protease treatment for GST tag cleavage. The arrow indicates the band at about 70 kDa position that remains unaltered by TEV protease treatment. (B) The ATPase activity staining of the purified p53 protein samples with and without TEV protease treatment. Both the lanes (-TEV and +TEV) contain 15 μg of purified p53 protein each for ATPase assay. The activity band in (B) aligns with the minor band indicated by an arrow in (A).

In order to map the ATPase activity with the protein band of p53, we performed the In-gel ATPase assay [21], wherein p53-GST protein was electrophoresed and allowed to recover the conformation in SDS-PAGE gel. The ATPase activity staining uncovered a band which did not coincide with the main full length p53-GST position (Figure 4B). This activity band aligned with 70 kDa standard marker rather than with the main p53-GST band (Compare Figure 4A and 4B). In spite of high protein level, p53 does not exhibit any detectable In-gel assay signal of ATP activity, while very low level of DnaK shows the activity in the same assay conditions (Figure 4 and 5). We excised the band that was showing the activity and performed LC-ESI-MS/MS for protein identification. The mascot search against the *H.sapien* and *E.coli* protein databases identified human protein p53 as well as E.coli proteins Chain A of Glutathione S transferase and Chaperone protein DnaK (Table 1). As observed upon coomassie staining, the activity band was just below the predominant p53-GST band, we suspected that p53 and GST might have come down as a smear from the main band. To eliminate this possibility, we cleaved the GST tag away from p53 using TEV protease, such that the activity band should be observed at untagged p53 position (∼53 kDa), if p53 indeed contributed to ATP hydrolysis. But In-gel ATPase assay revealed no change in the location of activity positive band even after TEV treatment (lane 3, Figure 5B), despite the clear migration of GST-free full length p53 at 53 kDa position (lane 2, Figure 5A). We excised the band that was showing the activity in post-TEV protease treated sample and analysed by LC-ESI-MS/MS. The band associated with ATPase activity was confirmed to be *E.coli* chaperone protein DnaK (Table 2). The enrichment of DnaK protein signals in the ATPase band from post-TEV protease treated sample was evident from the increased peptide coverage and mascot score (Compare Table 1 and Table 2). Proteins p53 and GST were absent in the activity band as shown by mass spectrometry.

**Table 1:** Identification of the In-gel ATPase activity stained band by LC-ESI-MS/MS analysis.

Host	Gene name	Mascot score	Peptide coverage	M.W.	Peptide hits
***H. sapien***	*p53*	2649	37%	44.1 kDa	MPEAAPPVAPAPAAPTPAAPAPAPSWPLSSSVPSQK
					LGFLHSGTA
					TCPVQLWVDSTPPPGTR
					CSDSDGLAPPQHLIRVEGNLRVEYLDDRNTFR
					RPILTIITLEDSSGNLLGR
					KKPLDGEYFTLQIR
					ELNEALELKDAQAGKEPGGSR
***E. coli***	*Chain A, Glutathione S Transferase*	14325	40%	32.2 kDa	LLLEYLEEKYEEHLYER
					KFELGLEFPNLPYYIDGDVK
					ERAEISMLEGAVLDIR
					IAYSKDFETLKVDFLSK
					RIAYSKDFETLKVDFLSK
					YIAWPLQGWQATFGGGDHPPK
	*Chaperone protein DnaK*	1438	28%	69.1 kDa	TTPSIIAYTQDGETLVGQPAKR
					QAVTNPQNTLFAIKR
					IIAADNGDAWVEVK
					TAEDYLGEPVTEAVITVPAYFNDAQR.
					IINEPTAAALAYGLDK
					LINYLVEEFKK
					AKLESLVEDLVNR
					VALQDAGLSVSDIDDVILVGGQTR
					KDVNPDEAVAIGAAVQGGVLTGDVK
					SLGQFNLDGINPAPR

P<0.05

The activity band shown in Figure 4(B) was excised and analyzed by LC-ESI-MS/MS. Table shows the peptide hits, peptide coverage and mascot scores for proteins identified in *H.sapien* and *E.coli* databases. The scores from the mascot search are significant with P<0.05.

**Table 2:** Identification of the In-gel ATPase activity stained band by LC-ESI-MS/MS analysis after GST cleavage by TEV protease.

Host	Gene name	Mascot score	Peptide coverage	M.W.	Peptide hits
***E. coli***	*Chaperone protein DnaK*	3621	57%	69.1 kDa	VLENAEGDRTTPSIIAYTQDGETLVGQPAKR
					QAVTNPQNTLFAIKR
					DVSIMPFK
					IIAADNGDAWVEVK
					MAPPQISAEVLKK
					TAEDYLGEPVTEAVITVPAYFNDAQR
					IAGLEVKR
					RIINEPTAAALAYGLDK
					LINYLVEEFKK
					DQGIDLR
					AKIELSSAQQTDVNLPYITADATGPK
					AKLESLVEDLVNR
					SIEPLKVALQDAGLSVSDIDDVILVGGQTR
					KDVNPDEAVAIGAAVQGGVLTGDVK
					HSQVFSTAEDNQSAVTIHVLQGER
					SLGQFNLDGINPAPR
					ASSGLNEDEIQK
					DAEANAEADRKFEELVQTR
					TAIESALTALETALKGEDK
					MQELAQVSQK
					LMEIAQQQHAQQQTAGADASANNAK

P<0.05

The activity band shown in Figure 5(B) Lane 3 was excised and analyzed by LC-ESI-MS/MS. Table shows the peptide hits, peptide coverage and mascot score for protein identified in *E.coli* database. The score from the mascot search is significant with P<0.05.

**Figure 6:**
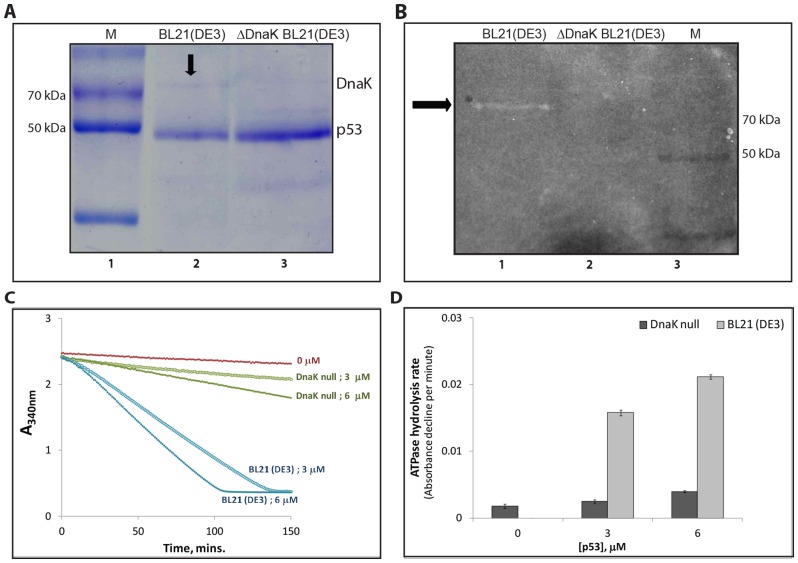
Absence of ATP hydrolysis activity in purified human p53 expressed in ΔDnaK BL21 (DE3) cells. (A) Coomassie stained SDS-PAGE showing purified p53 protein from BL21(DE3) (Lane 2) and ΔDnaK BL21 (DE3) *E.coli* cells (Lane 3) after GST tag removal. The *E.coli* protein, DnaK co-purifies with p53 as indicated by the arrow at about 70 kDa position (Marker, lane 1). The DnaK band is absent in p53 protein which was expressed and purified from DnaK null *E.coli* cells (Lane 3). (B) The ATPase activity staining of the p53 protein samples from DnaK containing (Lane 1) and DnaK null (Lane 2) *E.coli* cells. The activity band indicated by the arrow appears only in lane 1 which corresponds to DnaK at about 70 kDa position. Both the lanes (1 and 2) contain 12 μg of purified p53 protein each for the In-gel ATPase assay, which is three times more than in the coomassie stained gel (A). (C) Using the real time assay, we compared ATPase activity of human p53 purified BL21 (DE3) and ΔDnaK BL21 (DE3) *E.coli* cells. The p53 proteins were taken at two different concentrations of 3 and 6 μM. (D) Graph shows the rates of ATP hydrolysis in purified p53 proteins. ATPase rates were calculated as slopes of the plots in Figure (C). The graphs shown in (C) are a representative set from three independent experiments. Error bars in (D) indicate the standard deviation across triplicates of three independent experiments.

To further corroborate our results, we expressed and purified human p53 protein from a new host, ΔDnaK BL21(DE3) *E.coli* cells, that do not express DnaK protein. These cells are temperature sensitive and were grown at 30°C, instead of 37°C. As a control, we also expressed and purified human p53 protein from normal BL21(DE3) *E.coli* cells that expressed DnaK but were still cultured at 30°C. The coomassie stained SDS-PAGE gel revealed that the DnaK protein was co-purified with p53 from normal BL21(DE3) cells, but not from ΔDnaK BL21(DE3) *E.coli* cells (Figure 6A). We studied the ATPase activity in both these purified p53 proteins, using the In-gel ATPase assay (Figure 6B) and real time NADH dependent spectrophotometric assay (Figure 6C–D). The p53-GST protein was treated with TEV protease to cleave the GST tag prior to performing the assay, so as to clearly separate the DnaK (∼70 kDa) band from p53 (∼53 kDa) band on the gel. The In-gel assay showed the activity band at about 70 kDa position corresponding to DnaK only in the lane containing p53 protein purified from BL21(DE3) cells (Figure 6B, lane 1). This band was absent in the p53 purified from ΔDnaK host cells (Figure 6B, lane 2). Moreover, the activity band was not at the position of p53 protein in either of the lanes. These results were further confirmed by spectrophotometric assay where only the p53 protein purified from BL21(DE3) cells showed activity, whereas the one from ΔDnaK BL21(DE3) did not (Figure 6C–D).

*E.coli* DnaK is a homolog of eukaryotic chaperone, Hsp70 with overall homology of about 50% [24]. DnaK possesses an ATPase activity that was initially discovered because of its critical role in the DNA replication of bacteriophage [25]. Earlier studies had shown that specific association between Hsp70 and p53 is important for p53 chaperoning to support its tumor suppressor activity under stress conditions [26]. Similar to Hsp70, DnaK also forms a complex with p53 that dissociates in the presence of ATP [27]. Here we confirm that the ATP hydrolysis activity in purified p53 protein is due to the co-purification of one of its interactors DnaK from *E.coli*. We surmise that all the p53 deletion mutants 3C, 35, 25 and 24 showing varied ATPase activity (Figure 3) might be due to differential binding affinity with its interactor protein DnaK. Due to the promiscuous binding nature of p53, the likelihood of non specific interaction of overexpressed p53 with *E.coli* proteins is high. We conclude that the ATPase activity detected in purified p53 was entirely associated with DnaK, where p53 by itself does not possess ATPase activity. In lieu of this finding, we suggest that one has to be circumspect about the conclusions drawn on p53 functions based on its supposedly demonstrated ATPase activity. p53 interaction with DNA modulated by ATP/ADP must therefore be an indirect consequence of an interactor protein dependent ATPase activity. We strongly believe that a protein hub such as p53 that dynamically interacts with a large variety of cellular protein machineries might exhibit high propensity to facilitate co-purification of its strong interactor protein, compounding the biochemical characterization of intrinsic functions associated with p53 itself. The current study is an important alert in that direction, which is worth noting, given the “high profile” nature of p53 protein in higher eukaryotic cells.
